# Effects of physical activity and sleep duration on fertility: A systematic review and meta-analysis based on prospective cohort studies

**DOI:** 10.3389/fpubh.2022.1029469

**Published:** 2022-11-03

**Authors:** Fanqi Zhao, Xiang Hong, Wei Wang, Jingying Wu, Bei Wang

**Affiliations:** Key Laboratory of Environmental Medicine and Engineering of Ministry of Education, Department of Epidemiology and Health Statistics, School of Public Health, Southeast University, Nanjing, China

**Keywords:** physical activity, sleep duration, meta-analysis, fertility, review

## Abstract

**Objective:**

Subfertility is a common problem for couples in modern society. Many studies have confirmed that lifestyle factors can affect fertility although there are conflicting conclusions relating to the effects of physical activity and sleep duration on fertility. In this study, we aimed to summarize and analyze the available evidence.

**Methods:**

PubMed, Web of Science, Cochrane, and Embase databases (as of October 14, 2022) were systematically searched for eligible prospective cohort studies. Data were extracted and effect values were combined. We also performed methodological quality and bias risk assessments for all the included studies.

**Results:**

A total of 10 eligible articles were included in our analysis; seven investigated the relationship between physical activity and fertility, and three investigated the effect of sleep duration on fertility. Compared with the lowest level of physical activity, high intensity physical activity (the highest levels of physical activity) was negatively correlated with fertility [odds ratio (OR) = 0.84; 95% confidence interval (CI): 0.70, 1.00, *I*^2^ = 64%]. However, we did not find an association between moderate intensity physical activity and fertility (OR = 1.09; 95% CI: 0.98, 1.22, *I*^2^ = 60%). We observed an inverse association between limited sleep duration (≤ 7 h) and fertility (OR = 0.92; 95% CI: 0.84, 1.00, *I*^2^ = 0%) compared with 8 h of sleep. The relationship between long sleep duration (≥9 h) and fertility was not statistically significant (OR = 0.85; 95% CI: 0.60, 1.21, *I*^2^ = 83%). According to the Newcastle-Ottawa Scale score, the overall quality of the research articles included was ranked as medium to high (6–9). Through GRADE system, the quality of evidence for the impact of high intensity physical activity and limited sleep duration on fertility was moderate, while the quality of evidence for the impact of moderate intensity physical activity and long sleep duration on fertility was low.

**Conclusion:**

The current evidence shows that high intensity physical activity and limited sleep time are negatively related to fertility. But there was great heterogeneity among studies, and the quality of research evidence was low to median. Thus, further high-quality research is needed to confirm this conclusion.

**PROSPERO registration number:**

CRD42022298137.

## Background

Over the last few decades, the downtrend in fertility rates has become a worldwide public health problem (1–3). With fewer births and an aging population, social security systems in many countries are now under great strain (4). On the one hand, the decline in fertility is due to the fact that more and more people are unwilling to have children; on the other hand, many couples try hard but cannot achieve a successful pregnancy. A variety of lifestyle factors can have substantial effects on fertility, including caffeine, psychological stress, alcohol consumption, smoking, and an extremely low or high body mass index (BMI) (5).

Some epidemiological data show that obese women experience a longer time to conception, reduced conception rates and poor results when undergoing *in vitro* fertilization (6). Current research suggests that the best way to control obesity is to increase physical activity and reduce sedentary time, and to sleep for an appropriate amount of time. However, the evidence relating to the relationship between these health-related lifestyles and fertility is inconsistent among pregnancy-planning couples. Previous research involving competitive female athletes, identified a phenomenon referred to as menstrual cycle disorder, including oligomenorrhea and amenorrhea (7–11). In addition, high levels of physical activity are also associated with a longer menstrual cycle, delayed ovulation, a longer follicular phase (12), and a shorter luteal phase (13). It appears that intense physical activity may affect ovarian function and thus impair a woman's fertility. However, a previous study on ovulatory infertility found that a woman's risk of ovulatory infertility decreased with an increase of physical activity (14). Because previous studies have shown that moderate intensity physical activity has little effect on menstrual characteristics, and the health benefits of appropriate physical activity are well-known, some scientists believe that a certain level of physical activity will have a positive impact on fertility, but beyond a certain threshold, this may have a detrimental impact (15). Therefore, our epidemiological understanding of the relationship between the intensity of physical activity and fertility is still very limited.

Sleep is also an important lifestyle factor that can affect fertility. Some studies have shown that the relationship between sleep and physical activity is bi-directional (16) in that physical activity may improve sleep quality and sleep may promote more physical activity (17). Gaining an appropriate amount of sleep is necessary and beneficial for general well-being. A large number of studies have shown that limited or excessive sleep duration can lead to a variety of health problems, such as cardiovascular disease, metabolic disorders, stroke and even sudden death (18–21). Experts believe that 7–9 h of sleep per night is the most beneficial to health; those who sleep 7–9 h per night have the lowest risk of adverse health consequences (22). Over recent years, researchers have paid increasing levels of attention to the potential impact of sleep on reproductive health. Research has already shown that a short duration of sleep may lead to irregular menstruation and affect a woman's fertility (23). However, this association has yet to be fully established.

In the present study, we aimed to explore the impact of different intensities of physical activity and sleep duration on fertility so as to provide a certain basis for policy construction to enhance fertility and improve fertility status. Therefore, we performed a systematic review and meta-analysis based on human epidemiological studies to analyze and confirm the effects of physical activity and sleep duration on fertility.

## Methods

This study followed Preferred Reporting Items for Systematic Reviews and Meta-analysis (PRISMA) and was registered with PROSPERO (ID: CRD42022298137, https://www.crd.york.ac.uk/prospero/).

### Patient and public involvement

This study does not involve patient and public participation.

### Search procedure

In accordance with a prespecified study protocol, we searched four medical literature databases (PubMed, Web of Science, Cochrane, and Embase) to identify relevant studies from inception to the October 14, 2022. We used several combinations of terms such as “physical activity,” “exercise,” “sport,” “leisure activity,” “sedentary behavior,” “sleep,” “sleep time,” “sleep duration,” “fertility,” “fecundity,” “time to pregnancy,” and “subfertility” and adjusted these terms across different databases. In addition, we conducted a manual search of the reference sections of relevant papers to identify other studies that might exist. All articles were independently reviewed by two researchers.

### Criteria for inclusion

The inclusion criteria were as follows: (1) population: subjects who were ≥18 years-of-age and women had not yet reached menopause; (2) intervention: physical activity and sleep duration measured by self-report questionnaires, individual interviews, or objective tools; (3) comparison: the minimum exposure dose of physical activity and sleep duration is taken as the reference level; (4) outcome: the study described the relationship (odds ratio, OR) between different doses of exposure (physical activity or sleep duration) and natural fertility compared to the reference level; (5) study design: prospective cohort study. The language of the included articles is limited to English, without geographical restrictions.

### Criteria for exclusion

The exclusion criteria were as follows: (1) reviews, conference abstracts, comments, case reports, and animal studies; (2) the use of assisted reproductive technology or artificial intervention to conceive instead of natural methods; and (3) studies that evaluated physical activity as a continuous variable rather than a categorical variable.

### Study quality and bias risk assessment

The Newcastle Ottawa Scale (NOS) was used to evaluate the methodological quality of the included studies. The NOS scale evaluated the quality of each study from three aspects: selection of the study population, the comparability of study groups and outcome. The scale has an overall score of 9, with scores of 7–9 indicating high research quality, 5–6 indicating medium research quality, and a score below 5 indicating low research quality (24).

Two researchers conducted an independent assessment of the risk of bias common to cohort studies, including (1) selection bias: whether the subjects were representative; (2) attrition bias: whether the loss of follow-up had an impact on research results; (3) information bias: whether the measurement of exposure and outcome was accurate; (4) confounding bias: whether the confounding factors were fully adjusted; and (5) other forms of bias. The evaluation results were used to generate an infographic of bias risk to visually express the degree of bias risk for each article. These five aspects were described as a high risk of bias, a low risk of bias and an unclear risk of bias.

Use GRADEprofiler3.6 to evaluate the quality of evidence according to GRADE evaluation method. GRADE specifies four categories-high, moderate, low, and very low-that are applied to a body of evidence, the level of evidence represents the degree of recognition of the reliability of the evidence by the literature evaluator. GRADE first grades the evidence according to the type of study design. Randomized controlled trials are high-level evidence, observational studies are low-level evidence, and case reports and series of case observations are very low-level evidence. Then further evaluate the quality of evidence according to five reasons for degradation and three reasons for upgrading.

### Data extraction

Data were extracted independently by two researchers with appropriate qualifications, including first author, year of publication, study location, study population, age range of participants, number of participants, assessment tools for physical activity and sleep duration, fertility assessment methods, statistical analysis methodology and adjustment factors.

### Exposure dose

Physical activity (PA) was defined as “any physical activity resulting in energy expenditure caused by skeletal muscle contraction” (25). We classified total physical activity into three levels: the lowest level of physical activity, moderate intensity physical activity, and high intensity physical activity. A metabolic equivalent of task (MET), is a unit useful for describing the energy expenditure of a specific physical activity (26). Each activity category has a corresponding METs. The total metabolic equivalent per week can be obtained by multiplying the time spent in each category by the metabolic equivalent of task (MET) score and adding it. If the original author used the International Physical Activity Questionnaire-SF (IPAQ-SF) or baseline questionnaire to investigate the frequency, intensity and time of the participant's physical activity and calculated the total activity metabolic equivalent in manner that was divided according to the dose of metabolic equivalent per week, we took the highest dose as high intensity physical activity and the lowest dose as the lowest level of physical activity; intermediate doses were combined as moderate intensity physical activity. If the study divided physical activity into three levels according to metabolic equivalent, then moderate intensity physical activity was considered to be ≥500 MET-min/week while high intensity physical activity was considered to be ≥3,000 MET-min/week. If the metabolic equivalent of high-level physical activity in the original study did not meet these standards, then we combined it with medium level as moderate intensity physical activity. If the original study did not calculate the activity metabolic equivalent, but three classifications were made according to the characteristics of the participant's physical activity, then the three levels in the original study were taken as the three levels of our study. In general, high intensity physical activity refers to vigorous physical activity with high frequency or moderate physical activity for a long time every week for a period of time, which makes people feel tired and weak and has a certain impact on normal life, while moderate intensity physical activity will not affect normal life.

Studies have shown that 7–9 h of sleep is most appropriate for adults (22). Therefore, we considered 8 h of sleep as the reference level; ≤ 7 h was considered as too short and ≥9 h was considered as too long.

### Statistical analysis

The included studies were statistically analyzed by proportional hazards models or logistic regression models. Each study reported risk ratios (RRs) or ORs and 95% confidence intervals (95% CIs) for the multivariable adjusted model. We selected the fully adjusted model if there were multiple models. D'Agostino et al. (27) previously reported that the pooling of estimated RRs from proportional hazard models and logistic regression models was acceptable and accurate results can be obtained (25). The *I*^2^ statistic was used to assess the heterogeneity of the results reported in each study before statistical data were combined. If *I*^2^ < 50%, then the heterogeneity between studies was low and the fixed-effect model was used for data combination. If *I*^2^ > 50%, then there was a large level of heterogeneity between studies and the random-effect model applied for data combination (24). The combined effect values are shown in Forest plots. Publication bias was investigated using funnel plots and Egger' test. To explore heterogeneity, we performed subgroup analysis. For physical activity (PA), we investigated (1) the type of PA and (2) different geographical regions (continents). For sleep duration, we investigated (1) different genders and (2) different geographical regions (continents). All statistical analyses were performed using R4.1.1.

## Results

### Study selection

**Figure 1:**
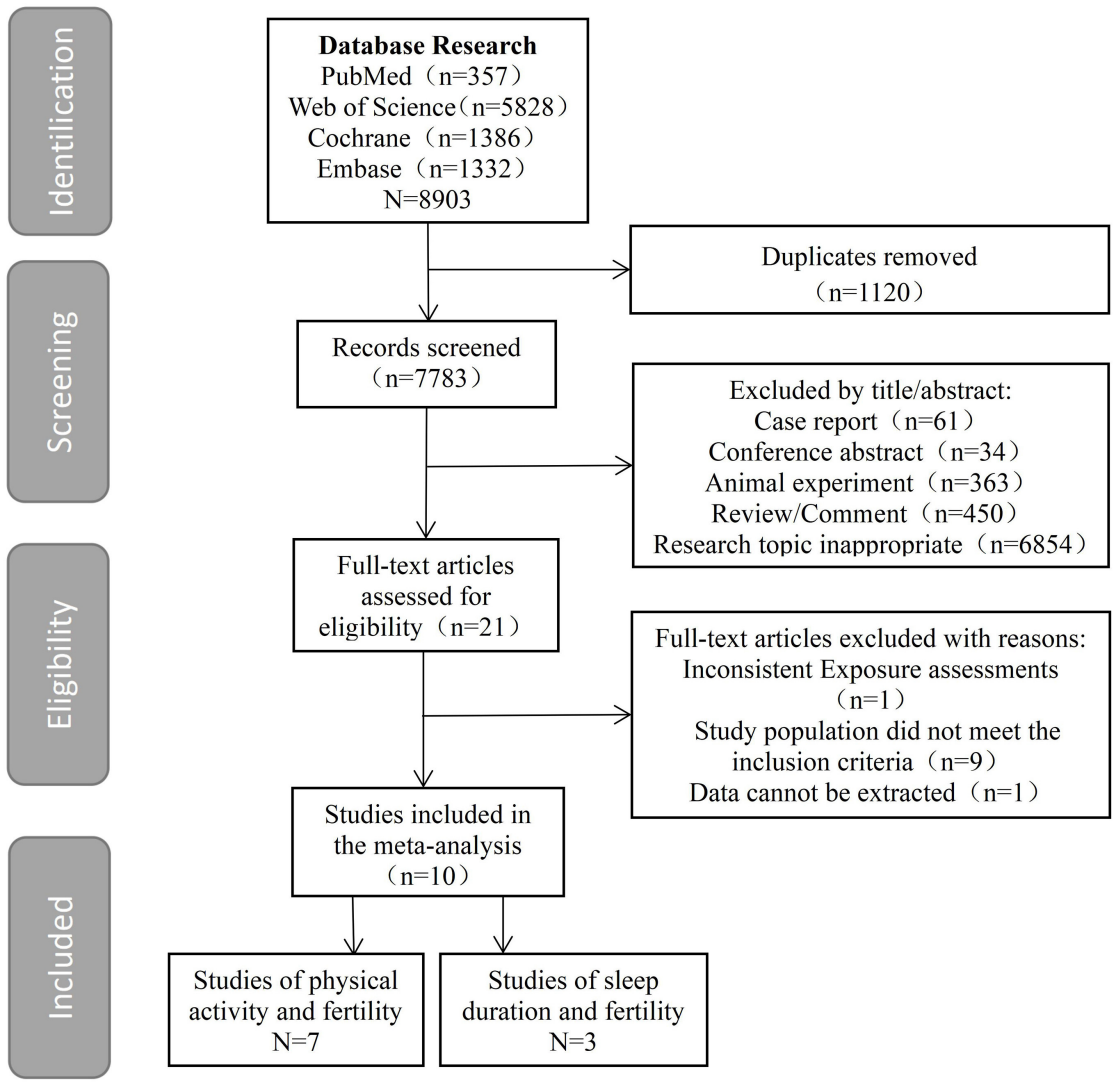
Selection process for the systematic review and meta-analysis.

A total of 8,903 potentially eligible articles were found by searching the four electronic databases; after removing duplicated articles, 7,783 remained. By reading titles and abstracts, we eliminated 7,762 articles and reviewed 21 articles in their entirety. After further screening, 10 articles were included in the meta-analysis, including seven articles relating to physical activity and fertility and three articles relating to sleep duration and fertility. Details of the selection process for studies is summarized in Figure 1.

### Studies and participant characteristics

The basic characteristics of the included studies are shown in Table 1. The study countries included the United States, Canada, the Netherlands, China, Norway, Denmark, Australia and Singapore. The study population came from eight cohorts. The age range of female participants was 18–49 years. There were 11 sets of results arising from 10 studies because one study reported both males and females. Only in the article of Florack (28), researchers obtained exposure information in the form of personal interviews; self-reported questionnaires were used to collect exposure information in other studies.

**Table 1:** Characteristics of selected studies.

**First author**	**Country**	**Study population**	**Sample size**	**Age range (years)**	**Exposure measure**	**Assessment of fertility**	**Statistical method**	**Adjustments**
Florack et al. (28)	Netherlands	Nonmedical female workers who were employed at 39 Dutch hospitals and were planning a pregnancy	259 (female)	18–39	Personal interview	Time to pregnancy (TTP)	Cox proportional hazards model	Exposure to detergents and vibration, working with video display terminals, suffering from chronic disease, partner's smoking, alcohol consumption and caffeine intake
Gudmundsdottir et al. (15)	Norway	Healthy premenopausal women participating in HUNT 1 and HUNT 2	3,887 (female)	20–45	Self-reported questionnaire	TTP	Logistic regression	Age, parity, smoking and marital status
Wise (29)	Danish	Women planning a pregnancy in the ‘Snart Gravid' Study	3,628 (female)	18–40	Self-reported questionnaire	TTP	Cox proportional hazards model	Cycle number, age, partner's age, BMI, alcohol consumption, pack-years of smoking, intercourse frequency, and last method of contraception
McKinnon et al. (30)	United States, Canada	Pregnancy planners in the Pregnancy Study Online (PRESTO)	2,062 (female)	21–45	Self-reported questionnaire	TTP	Proportional probabilities models	Age, parity, intercourse frequency, education, BMI, race/ethnicity, household income, marital status, last method of contraception, alcohol consumption, smoking and partner BMI
Russo et al. (31)	United States	Women with a history of one to two prior documented miscarriages	1,128 (female)	18–40	Self-reported questionnaire	TTP	Cox proportional hazards models	Marital status, parity and BMI
Mena et al. (32)	Australian	Women,from Australian Longitudinal Study on Women's Health (ALSWH), had tried to conceive or become pregnant on at least one survey between 2000 and 2015	6,130 (female)	18–42	Self-reported questionnaire	Problems with fertility	Cox proportional hazards regression	Age, marital status, country of birth and highest qualifification
Loy et al. (33)	Singapore	Asian women of Chinese, Malay or Indian ethnicity attempting to conceive within the next 12 months	523 (female)	18–45	Self-reported questionnaire	TTP	Discrete-time proportional hazards model	Age, ethnicity, education and parity, physical activity, body mass index, probable anxiety, probable depression
Wise (34)	United States, Canada	Male partners of those who planned to become pregnant in the Pregnancy Study Online (PRESTO)	1,083 (male)	≥21	Self-reported questionnaire	TTP	Proportional probabilities regression models	Male and female age, male and female BMI, female partner's sleep duration, intercourse frequency, and the following factors for males: depression, anxiety disorder, hypertension, diabetes, gastroesophageal reflux disease, race/ethnicity, education, use of multivitamins or folate supplements, smoking history, employment status, hours of work, hours of laptop use on one's lap, physical activity, caffeine intake, alcohol intake, sugar-sweetened soda intake, perceived stress scale (PSS-10), and previously fathered a child
Willis et al. (19)	United States, Canada	Pregnancy planners in the pregnancy study online (PRESTO)	1,743 (female)	21–45	Self-reported questionnaire	TTP	Proportional probabilities regression models	Female age, BMI, income, non-Hispanic white, prior birth, prior form of hormonal birth control, current smoker, hours working, history of infertility, unemployment, and caffeine consumption
Shi et al. (35)	China	Woman in CHNS: China Health and Nutrition Survey	2,687 (female)	18–49	Self-reported questionnaire	Probability of conception	Logistic regression	Age, race, working status, BMI, marital status, contraception, and caring for children, and the following factors for males: age, sleep duration, race, working status, BMI, and smoking status
Shi et al. (35)	China	The spouse of a woman in CHNS: China Health and Nutrition Survey	2,252 (male)	Interquartile range: 33–44	Self-reported questionnaire	Probability of conception	Logistic regression	Age, race, working status, BMI, smoking status, and the following factors for females: age, sleep duration, race, working status, BMI, contraception, and caring for children

### Literature quality and risk of bias

**Figure 2:**
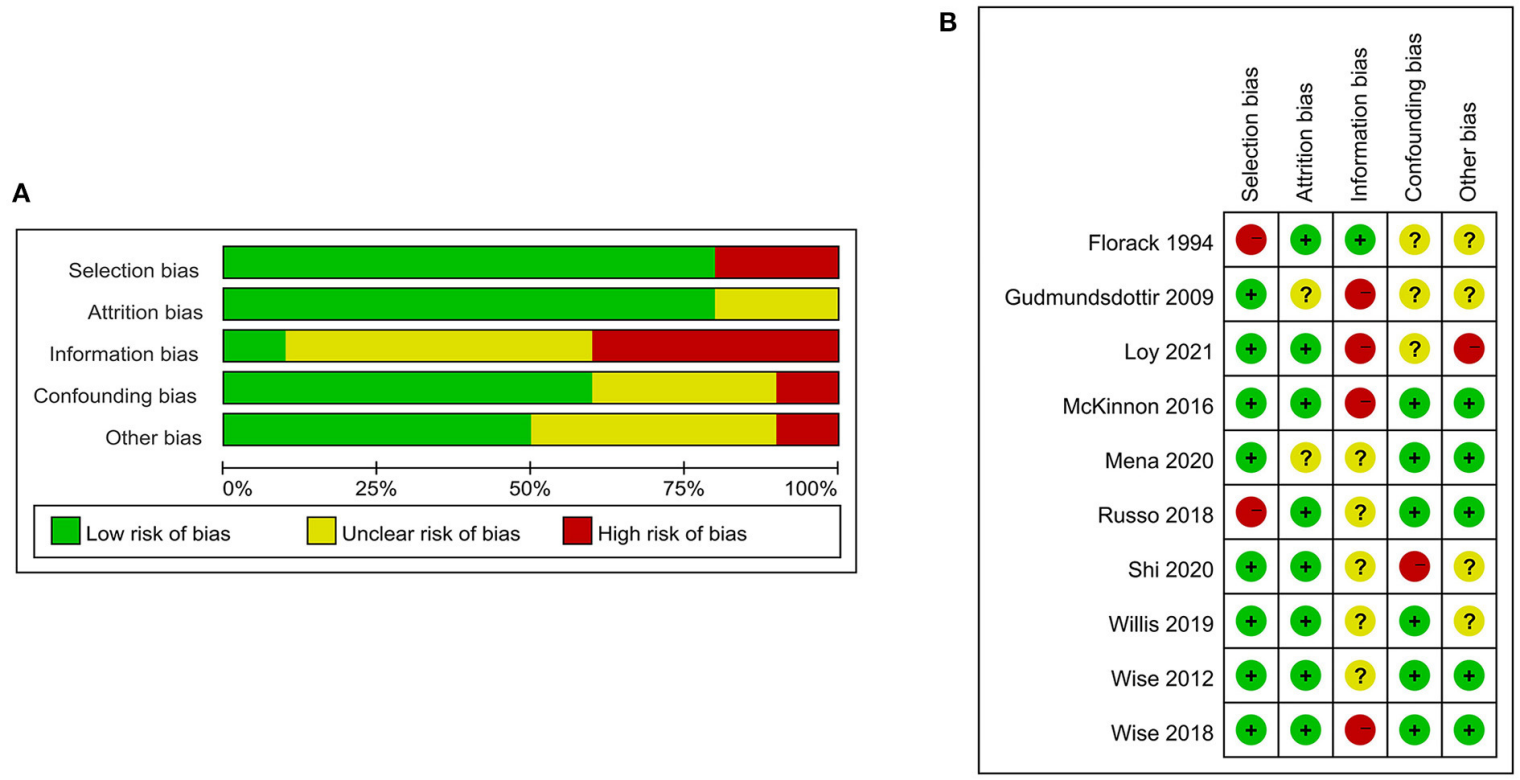
Bias risk assessment of 10 studies included in the meta-analysis.

Nine of the 10 articles had a quality score of 7–9; only one of these had a quality score of 6 on the Newcastle-Ottawa Scale (Supplementary Table 1), thus representing moderate to high methodological quality. Some studies had a higher risk of information bias because information relating to exposure and fertility was self-reported by the participants. In addition, some variables were not available, leading to some uncontrollable confounding bias (Figure 2).

According to the GRADEprofiler rating results (Supplementary Figures 1, 2), the quality of research evidence on the impact of high intensity physical activity and limited sleep duration on fertility is moderate, while the quality of research evidence on the impact of moderate intensity physical activity and long sleep duration on fertility is low.

### Effects of PA on fertility

**Figure 3:**
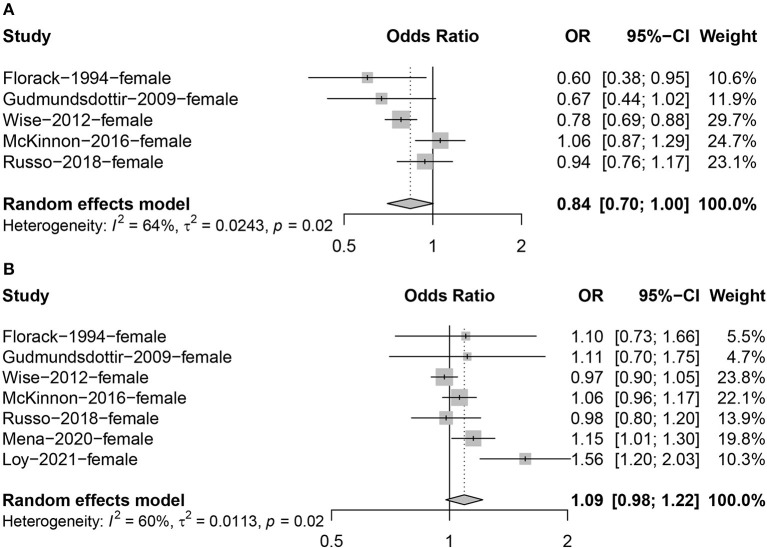
Forest plot of the combined effect size of the effects of high **(A)** and moderate **(B)** intensity physical activity on fertility compared to the lowest levels of physical activity.

We evaluated seven articles relating to the association between total physical activity levels and fertility, of which five reported the effect of high intensity physical activity on fertility and seven reported the effect of moderate intensity physical activity on fertility. Due to the large heterogeneity between studies when combining the effect values of physical activity (high intensity PA: *I*^2^ = 64%, *P* = 0.02; moderate intensity PA: *I*^2^ = 60%, *P* = 0.02; Figure 3), we chose the random effect model. Meta-analysis showed that high intensity physical activity was inversely associated with fertility in the study population compared to the lowest levels of physical activity (OR = 0.84; 95% CI: 0.70, 1.00; Figure 3A). No association was found between moderate intensity physical activity and fertility (OR = 1.09; 95% CI: 0.98, 1.22; Figure 3B).

### Effect of sleep duration on fertility

**Figure 4:**
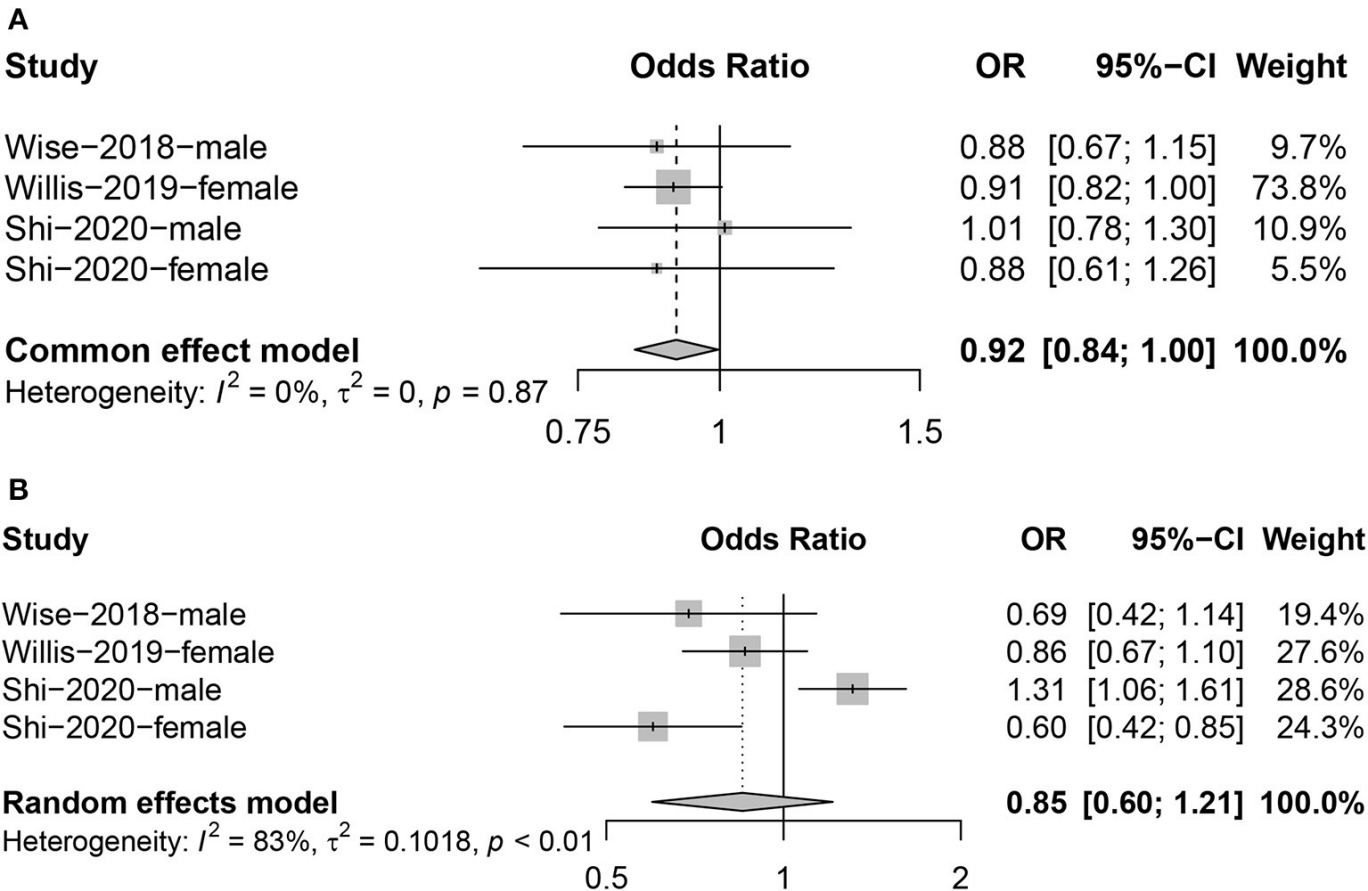
Forest plot of the combined effect size of the effects of too short [**(A)**: ≤ 7 hours] or too long [**(B)**: ≥ 9 hours] sleep duration on fertility compared to 8 hours.

We included three articles investigating the relationship between sleep duration and fertility, with a total of four results, as one of the articles reported results for both male and female populations. Meta-analysis found a negative correlation between limited sleep duration (≤ 7 h) and fertility (OR = 0.92; 95% CI: 0.84, 1.00; Figure 4A); the heterogeneity between studies was very small (*I*^2^ = 0%, *P* = 0.87). However, we found no evidence that a long sleep duration (≥9 h) had the same impact (OR = 0.85; 95% CI: 0.60, 1.21, *I*^2^ = 83%; Figure 4B).

### Subgroup and sensitivity analysis

Subgroup analysis of the types of physical activity found that the high intensity physical activity group of leisure physical activity was negatively correlated with fertility. High intensity physical activity in Europe was also inversely associated with fertility. In the subgroup analysis of sleep duration, we found an inverse association between men who slept ≥9 h and fertility, and there is a negative correlation between short sleep duration (≤ 7 h) and fertility in North America. Specific information relating to the subgroup analysis is shown in Table 2.

**Table 2:** Subgroup analysis.

**Subgroup analysis**	**OR (95%CI)**	***I*^2^ (%)**	**Number of cohorts**	***P*-value**
	**PA**			
**Type of PA**
High intensity PA				
Leisure PA	0.77 (0.68, 0.87)	0	2	< 0.0001
Occupational PA	0.60 (0.40, 1.00)	0	1	
Both	1.00 (0.87, 1.16)	0	2	0.9475
Moderate intensity PA			
Leisure PA	1.05 (0.91, 1.21)	62	3	0.4914
Occupational PA	1.10 (0.70, 1.60)	0	1	
Both	1.15 (0.89, 1.49)	76	3	0.2751
**Continent**
High intensity PA				
Europe	0.76 (0.67, 0.85)	0	3	< 0.0001
North America	1.00 (0.87, 1.16)	0	2	0.9475
Moderate intensity PA				
Europe	0.98 (0.91, 1.05)	0	3	0.5511
North America	1.04 (0.96, 1.14)	0	2	0.3411
Oceania	1.15 (1.01, 1.30)	0	1	
Asia	1.56 (1.20, 2.04)	0	1	
	**Sleep duration**			
**Sex**
≤7 h				
Male	0.88 (0.71, 1.09)	0	2	0.2456
Female	0.92 (0.84, 1.01)	0	2	0.0835
≥9 h				
Male	0.63 (0.47, 0.83)	0	2	0.0014
Female	1.07 (0.71, 1.61)	85	2	0.7595
**Continent**
≤7 h				
North America	0.91 (0.83, 0.99)	0	2	0.0369
Asia	0.96 (0.78, 1.19)	0	2	0.7317
≥9 h				
North America	0.82 (0.66, 1.03)	0	2	0.0836
Asia	0.90 (0.42, 1.93)	93	2	0.7826

### Publication bias

For publication bias, the funnel plot was relatively symmetrical (Supplementary Figures 3, 4); no significant publication bias was detected by Egger' test (*P* > 0.05).

## Discussion

This meta-analysis showed that high intensity physical activity and limited sleep duration were negatively correlated with fertility.

A retrospective case-control study found that vigorous exercise for an hour or more a day increased the risk of infertility by 6.2-fold (36); therefore, it appeared that strenuous exercise will have a negative impact on female fertility. However, a study in a large cohort of American women found that increased levels of vigorous activity were associated with a reduced relative risk of ovulatory infertility (37); the same conclusion was reached in another study investigating lifestyle and ovulatory infertility (14). However, these two articles focused on ovulatory infertility. Ovulatory infertility is only one type of infertility and cannot fully represent female fertility; therefore, these research results are not widely representative. In addition, we found that most studies on men evaluated sperm quality rather than directly assessing the relationship between fertility and physical activity so we did not include this information in our study. Although it is generally believed that appropriate physical activity is beneficial to physical health, we did not find a statistically significant association between moderate intensity physical activity and improved fertility.

The mechanism by which high intensity physical activity might impair fertility may be related to disorders of the female menstrual cycle and the decline of male sperm quality. Studies have shown that hypothalamic amenorrhea occurs because excessive exercise leads to a lack of energy in the body, thus affecting levels of estrogen through the hypothalamic pituitary ovarian axis, leading to a long-term decrease in circulating estradiol levels and a reduction in ovarian stimulation (38). Amenorrhea is one of the dysfunction of “female athletes triad,” compared with a sedentary control group, athletes are more prone to changes in the menstrual cycle; the secretion of corticotropin releasing hormone (CRH) increased in women with changes in menstrual cycle, and CRH inhibite the release of gonadotropin releasing hormone (GnRH) which inhibit the normal pulsatile secretion mode of gonadotropin (39). For men, excessive exercise can reduce the function of hypothalamus pituitary gonad axis (HPT), increase oxidative stress and chronic inflammation, resulting in decreased semen quality, thus impairing male fertility (40). Semen evaluation of male athletes found that sperm DNA fragmentation was affected by high level sports training (41). Male cyclists were also found to have alterations in the morphology of their sperm (42). Another study of young, healthy men found that more active men had a higher proportion of inactive sperm (43). Although excessive exercise impairs fertility, lack of exercise also leads to impaired reproductive function, the most common of which is polycystic ovary syndrome caused by obesity due to lack of exercise. Therefore, appropriate exercise is necessary, which can improve the hormone level, menstrual cycle, ovulation function of women and semen quality of men.

Sleep deprivation is becoming a common health problem in modern society (44); a growing number of studies have shown an association between insufficient sleep and decreased fertility (45). The mechanism by which sleep duration can act on the reproductive system is closely related to circadian rhythm (46). Many hormones are involved in reproductive function, such as thyroid stimulating hormone, luteinizing hormone and testosterone; these are all regulated by circadian rhythm (47). If the circadian rhythm is disordered, then there will be an inevitable effect on the levels of reproductive hormones (43, 48). Sleep deprivation induces stress responses by activating the hypothalamus-pituitary-adrenal (HPA) axis, thereby disrupting the levels of various reproductive hormones (49). When reproductive hormone levels are abnormal, ovulation may not be stimulated. In addition, studies have shown that a short sleep duration can also lead to irregular menstrual cycles (50) *via* the probable action of hormones. TSH levels increase during sleep, while acute sleep deprivation in healthy young women in the follicular phase have been associated with a significant increase in TSH levels (51, 52). High levels of TSH can lead to irregular menstruation and even amenorrhea. Some studies speculate that insufficient sleep may affect women's fertility through impaired immunity (47). During sleep deprivation, increased cytokine and immune inflammatory reaction marked by TNF and IL-6 were observed (53–55), high levels of IL-6 and TNF were considered to be related to female infertility (56, 57). In a previous study of males, it was found that a short sleep duration was associated with a reduction in semen quality (58–61). The number of anti-sperm antibodies in the semen is known to increase with a short sleep duration. These antibodies destroy healthy sperm and sperm quality (50). In our meta-analysis, the association between a long sleep duration and fertility was not statistically significant. However, in a longitudinal observational study of male college students in Chongqing, China, it was found that excessive sleep may also damage semen quality (62). More high-quality studies are needed to explore the effects of long sleep duration on reproductive function.

Our research had certain advantages. All the studies we included were prospective cohort studies; therefore, these studies were effective for inferring causality. Furthermore, the sample size of the studies we included was large and the sample representation was good. We have summarized the current evidence relating to the effects of physical activity and sleep duration on fertility, thus providing a scientific basis for further research on the factors that influence fertility. Our findings can also guide pregnancy-planning couples to adjust their lifestyle appropriately to improve their fertility. However, there were also some limitations in our meta-analysis that need to be considered. First, all of the included studies included measurements of exposure obtained through self-reported questionnaires or personal interviews, rather than objective measurement. The content of self-reports may include recall bias, which may lead to inconsistency and inaccuracy of the measurement results. Second, our division of physical activity intensity was not quantitative because not all of the original articles used metabolic equivalents. Furthermore, there were notable differences in the definition of physical activity intensity between different studies; therefore, it was not clear how strong high intensity physical activity actually is, although our research revealed a negative correlation between the highest level of physical activity and fertility. In addition, high levels of heterogeneity were detected in studies of the effects of physical activity and long sleep duration on fertility, possibly due to differences in race and exposure measurements, so we used a random effects model and performed subgroup analysis to reduce the effects of heterogeneity. Due to the wide existence of covariates, although we included an adjusted model, we still need to consider confounding bias.

Whether for reproductive function or other physical functions, a good lifestyle is necessary for people. Moderate physical activity and sufficient sleep are beneficial to physical and mental health, while high intensity physical activity and limited sleep duration will consume people's energy and affect their normal life. Therefore, we suggest that pregnancy-planning couples should properly adjust their lifestyle, carry out moderate physical activities within the scope of their ability, avoid high-frequency violent activities, and ensure sufficient sleep time and good sleep quality, so as to improve the fertility. In the future, we need more high quality studies that use objective methods to measure exposure and fully adjust for confounding factors to confirm our conclusions and determine the threshold for high intensity physical activity. In addition, further research is needed to explore the mechanisms underlying the effects of physical activity and sleep duration on fertility so as to provide a scientific basis for improving fertility.

## Conclusion

Among the known lifestyle factors that affect fertility, high intensity physical activity and limited sleep duration are negatively correlated with fertility. But there was great heterogeneity among studies, and the quality of research evidence was low to moderate. Further research is needed to confirm this conclusion and study the mechanisms involved.

## Data availability statement

The original contributions presented in the study are included in the article/Supplementary material, further inquiries can be directed to the corresponding author.

## Author contributions

FZ and XH originally designed the idea of the study and assessed the methodological quality of the studies met the inclusion criteria. FZ did the analysis for the study and wrote the initial draft. WW and JW contributed to the amendment of the manuscript and suggestions for data analysis. XH was responsible for revising it critically for important intellectual content. BW was responsible for reviewing all drafts of the manuscript. All authors read and approved the final manuscript.

## Funding

This research was supported by National Natural Science Foundation of China (Nos. 81872634 and 82204057); Natural Science Foundation of Jiangsu Province, China (No. BK20220827); and the Scientific research project of Jiangsu Provincial Health Commission (No. ZD2021047).

## Conflict of interest

The authors declare that the research was conducted in the absence of any commercial or financial relationships that could be construed as a potential conflict of interest.

## Publisher's note

All claims expressed in this article are solely those of the authors and do not necessarily represent those of their affiliated organizations, or those of the publisher, the editors and the reviewers. Any product that may be evaluated in this article, or claim that may be made by its manufacturer, is not guaranteed or endorsed by the publisher.
