# Supplementary material for: Effects of physical activity and sleep duration on fertility: A systematic review and meta-analysis based on prospective cohort studies
Source: Front Public Health. 2022 Nov 3;10:1029469. doi: 10.3389/fpubh.2022.1029469 (PMC9669984; doi:10.3389/fpubh.2022.1029469)
Supplement: Supplementary file 1 [file Table_1.DOCX]

**Supplementary material content**

**Supplementary material 1. Search term and results**

**Supplementary Table 1. The Newcastle-Ottawa Scale (NOS) used to assess the quality of the 10 studies included in the meta-analysis**

**Supplementary Figure 1. Quality of evidence on the effects of high intensity physical activity and moderate intensity physical activity on fertility**

**Supplementary Figure 2. Quality of evidence on the effects of too short (≤7hours) and too long (≥9hours) sleep duration on fertility**

**Supplementary Figure 3. The funnel plot for the association between high (A) and moderate (B) intensity physical activity and fertility**

**Supplementary Figure 4. The funnel plot for the association between too short (A:≤7hours) and too long (B:≥9hours) sleep duration and fertility**

**Supplementary material 1. Search term and results**

**Search term:** (((sleep duration[Title/Abstract]) OR (sleep time[Title/Abstract])) OR (("Sleep"[Mesh]) OR ((((((((Sleeping Habits[Title/Abstract]) OR (Sleep Habits[Title/Abstract])) OR (Habit, Sleep[Title/Abstract])) OR (Habits, Sleep[Title/Abstract])) OR (Sleep Habit[Title/Abstract])) OR (Sleeping Habit[Title/Abstract])) OR (Habit, Sleeping[Title/Abstract])) OR (Habits, Sleeping[Title/Abstract])))) OR ((((((((Sport[Title/Abstract]) OR (Athletics[Title/Abstract])) OR (Athletic[Title/Abstract])) OR ("Sports"[Mesh])) OR ((((((Activities, Leisure[Title/Abstract]) OR (Activity, Leisure[Title/Abstract])) OR (Leisure Activity[Title/Abstract])) OR (Leisure[Title/Abstract])) OR (Leisures[Title/Abstract])) OR ("Leisure Activities"[Mesh]))) OR (("Exercise"[Mesh]) OR (((((((((((((((((((((((((Exercises[Title/Abstract]) OR (Physical Activity[Title/Abstract])) OR (Activities, Physical[Title/Abstract])) OR (Activity, Physical[Title/Abstract])) OR (Physical Activities[Title/Abstract])) OR (Exercise, Physical[Title/Abstract])) OR (Exercises, Physical[Title/Abstract])) OR (Physical Exercise[Title/Abstract])) OR (Physical Exercises[Title/Abstract])) OR (Acute Exercise[Title/Abstract])) OR (Acute Exercises[Title/Abstract])) OR (Exercise, Acute[Title/Abstract])) OR (Exercises, Acute[Title/Abstract])) OR (Exercise, Isometric[Title/Abstract])) OR (Exercises, Isometric[Title/Abstract])) OR (Isometric Exercises[Title/Abstract])) OR (Isometric Exercise[Title/Abstract])) OR (Exercise, Aerobic[Title/Abstract])) OR (Aerobic Exercise[Title/Abstract])) OR (Aerobic Exercises[Title/Abstract])) OR (Exercises, Aerobic[Title/Abstract])) OR (Exercise Training[Title/Abstract])) OR (Exercise Trainings[Title/Abstract])) OR (Training, Exercise[Title/Abstract])) OR (Trainings, Exercise[Title/Abstract]))))) OR (("Sedentary Behavior"[Mesh]) OR ((((((((((Behavior, Sedentary[Title/Abstract]) OR (Sedentary Behaviors[Title/Abstract])) OR (Sedentary Lifestyle[Title/Abstract])) OR (Lifestyle, Sedentary[Title/Abstract])) OR (Physical Inactivity[Title/Abstract])) OR (Inactivity, Physical[Title/Abstract])) OR (Lack of Physical Activity[Title/Abstract])) OR (Sedentary Time[Title/Abstract])) OR (Sedentary Times[Title/Abstract])) OR (Time, Sedentary[Title/Abstract])))) AND ((("Time-to-Pregnancy"[Mesh]) OR ((Time to Pregnancy[Title/Abstract]) OR (Time-to-Pregnancies[Title/Abstract]))) OR ((("Fertility"[Mesh]) OR (((((((((((((((((((((((((((Fecundability[Title/Abstract]) OR (Fecundity[Title/Abstract])) OR (Differential Fertility[Title/Abstract])) OR (Fertility, Differential[Title/Abstract])) OR (Fertility Determinants[Title/Abstract])) OR (Determinant, Fertility[Title/Abstract])) OR (Determinants, Fertility[Title/Abstract])) OR (Fertility Determinant[Title/Abstract])) OR (Subfecundity[Title/Abstract])) OR (Fertility Preferences[Title/Abstract])) OR (Fertility Preference[Title/Abstract])) OR (Preference, Fertility[Title/Abstract])) OR (Preferences, Fertility[Title/Abstract])) OR (Fertility, Below Replacement[Title/Abstract])) OR (Below Replacement Fertility[Title/Abstract])) OR (Marital Fertility[Title/Abstract])) OR (Fertility, Marital[Title/Abstract])) OR (Natural Fertility[Title/Abstract])) OR (Fertility, Natural[Title/Abstract])) OR (World Fertility Survey[Title/Abstract])) OR (Fertility Survey, World[Title/Abstract])) OR (Fertility Surveys, World[Title/Abstract])) OR (Survey, World Fertility[Title/Abstract])) OR (Surveys, World Fertility[Title/Abstract])) OR (World Fertility Surveys[Title/Abstract])) OR (Fertility Incentives[Title/Abstract])) OR (Fertility Incentive[Title/Abstract])))))

**Search results:** A total of 8903 documents were retrieved from four databases, including 357 in PubMed, 5828 in Web of Science, 1386 in Cochrane Library and 1332 in Embase.

**Supplementary Table 1. The Newcastle-Ottawa Scale (NOS) used to assess the quality of the 10 studies included in the meta-analysis**

| Author (Year) | Selection | Comparability | Outcome | Total |
| --- | --- | --- | --- | --- |
| Florack (1994) | ★★★ | ★★ | ★★★ | 8 |
| Gudmundsdottir (2009) | ★★★ | ★ | ★★★ | 7 |
| Wise (2012) | ★★★ | ★★ | ★★★ | 8 |
| McKinnon (2016) | ★★★ | ★★ | ★★★ | 8 |
| Russo (2018) | ★★ | ★ | ★★★ | 6 |
| Wise (2018) | ★★★ | ★★ | ★★★ | 8 |
| Willis (2019) | ★★★ | ★★ | ★★★ | 8 |
| Mena (2020) | ★★★ | ★ | ★★★ | 7 |
| Shi (2020) | ★★★ | ★★ | ★★★ | 8 |
| Loy (2021) | ★★★★ | ★★ | ★★★ | 9 |


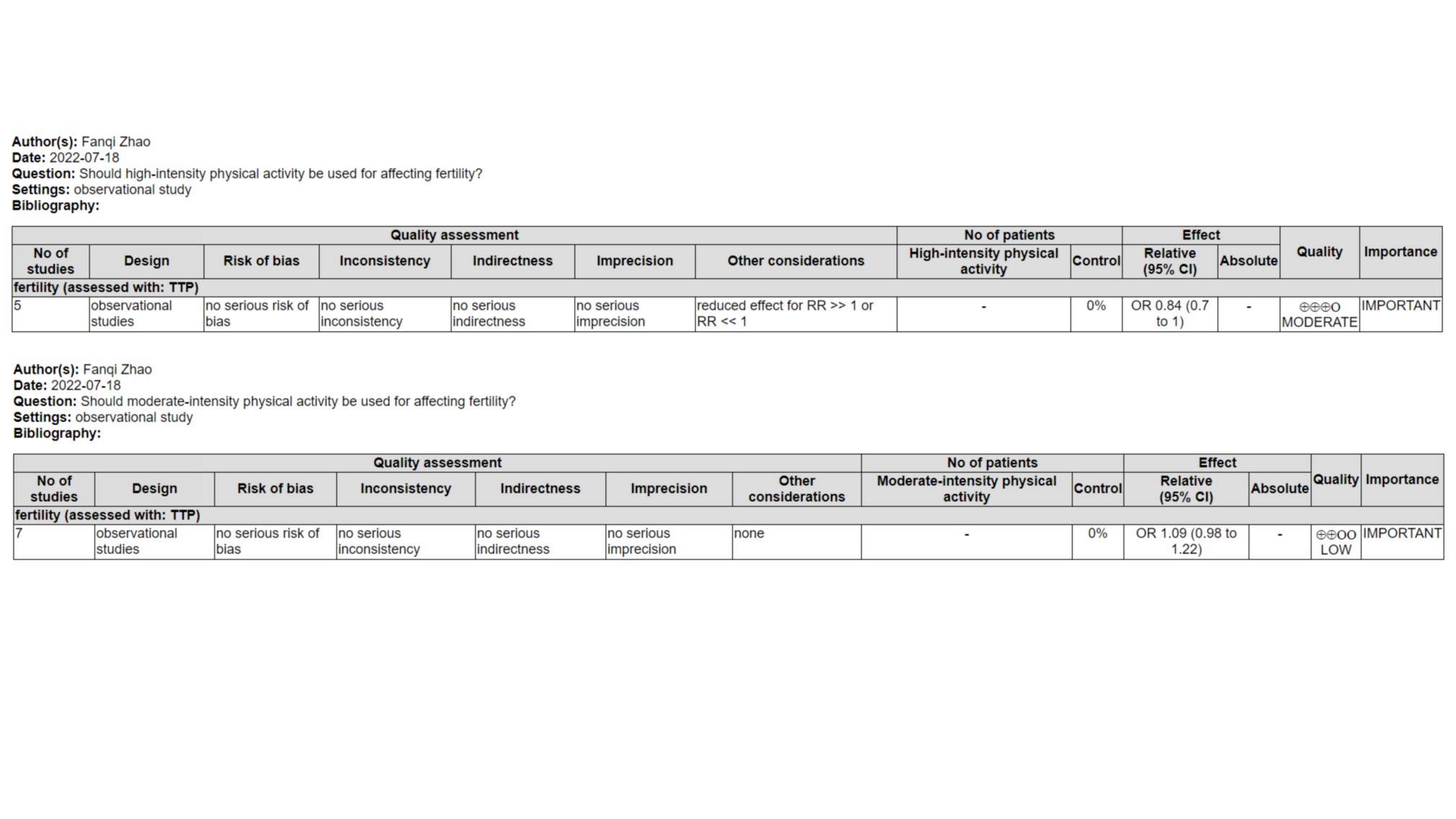


**Supplementary Figure 1.** **Quality of evidence on the effects of high intensity physical activity and moderate intensity physical activity on fertility**


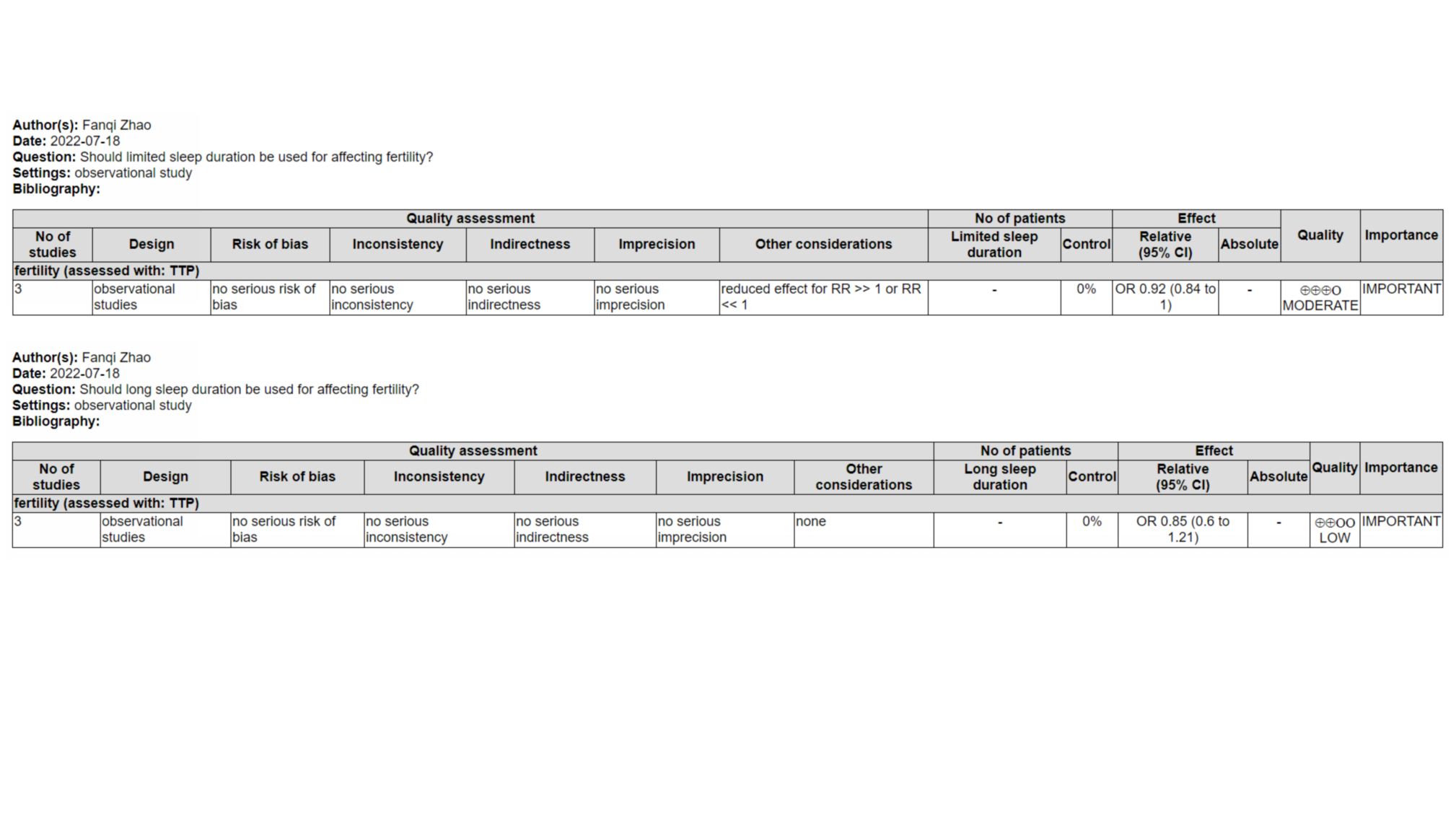


**Supplementary Figure 2.** **Quality of evidence on the effects of too short (≤7hours) and too long (≥9hours) sleep duration on fertility**


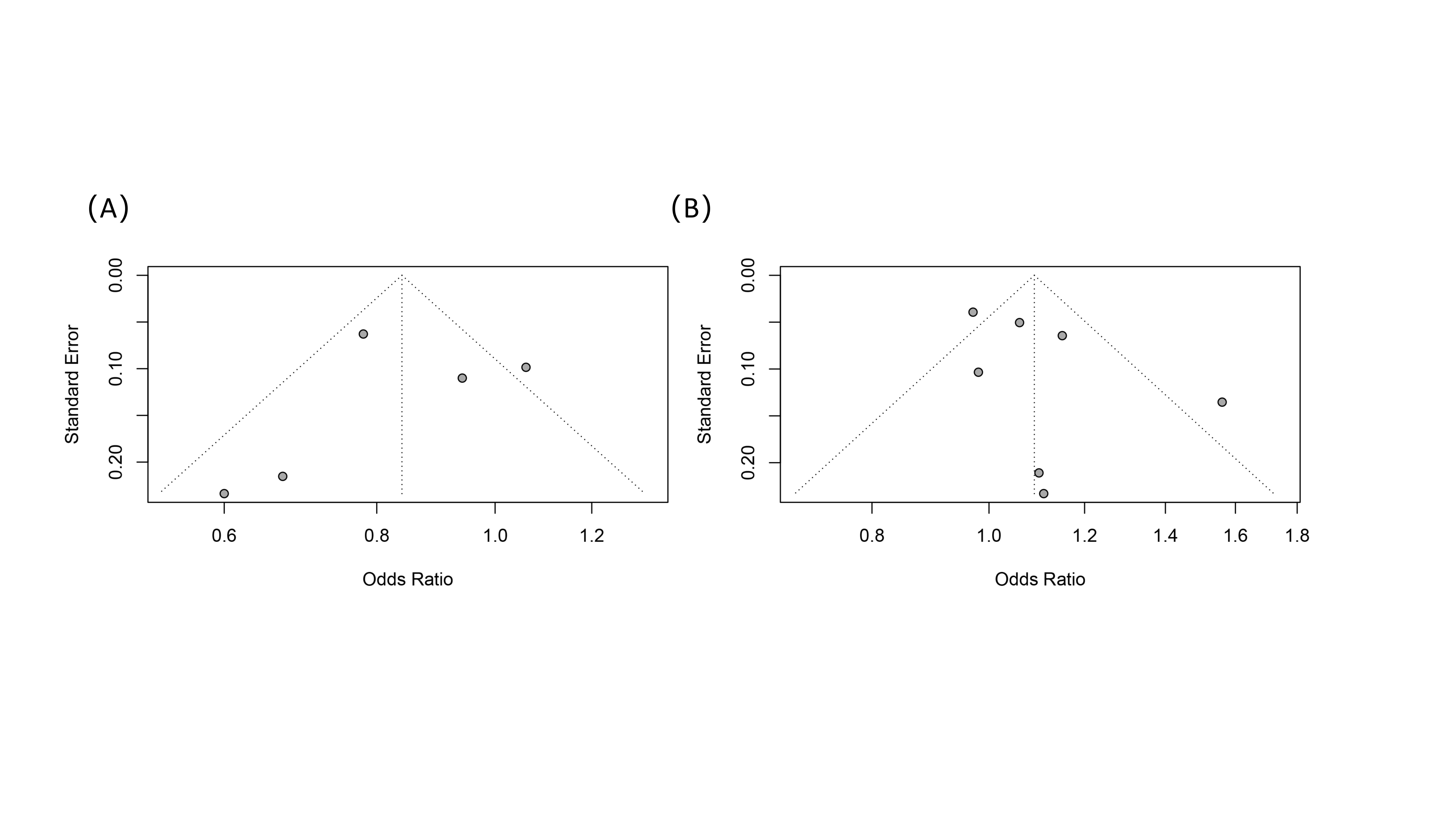


**Supplementary Figure 3.** **The funnel plot for the association between high (A) and moderate (B) intensity physical activity and fertility**

**
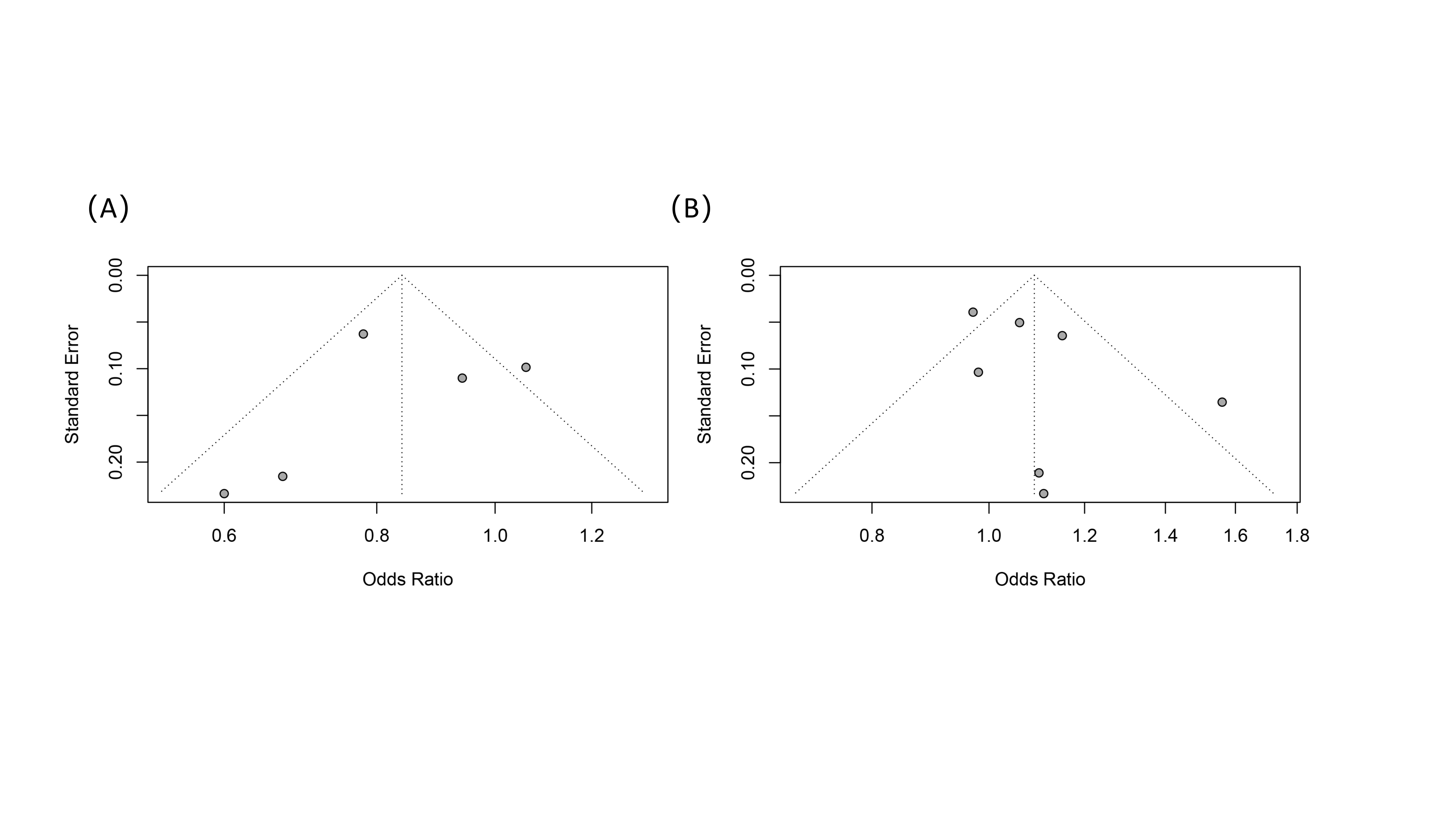
**

**Supplementary Figure 4. The funnel plot for the association between too short (A:≤7hours) and too long (B:≥9hours) sleep duration and fertility**
